# Novel insect-based child nutrition: the position of the nutritional committee of the Italian society of pediatric gastroenterology, hepatology and nutrition (SIGENP)

**DOI:** 10.1186/s13052-023-01565-x

**Published:** 2023-12-01

**Authors:** Lorenzo Norsa, Carlo Agostoni, Teresa Capriati, Angelo Campanozzi, Antonella Diamanti, Antonella Lezo, Paolo Gandullia, Maria Immacolata Spagnuolo, Claudio Romano

**Affiliations:** 1 Pediatric Hepatology, Gastroenterology and Transplantation ASST Papa Giovanni XXIII, Piazza Oms 1, 24127 Bergamo, Italy; 2 Pediatric Unit, Fondazione IRCCS Ca’ Granda Ospedale Maggiore Policlinico, University of Milan, Milan, Italy; 3 Gastroenterology and Nutritional Rehabilitation, Bambino Gesù Children’s Hospital, Rome, Italy; 4 Pediatrics, Department of Medical and Surgical Sciences, University of Foggia, Foggia, Italy; 5 Dietetic and Clinical Nutrition Unit, Pediatric Hospital Regina Margherita, University of Turin, Turin, Italy; 6 Pediatric Gastroenterology and Endoscopy Unit, Institute “Giannina Gaslini”, Genoa, Italy; 7 Department of Translational Medical Sciences - Section of Pediatrics, University of Naples “Federico II”, Naples, Italy; 8 Pediatric Gastroenterology and Cystic Fibrosis Unit, Department of Human Pathology in Adulthood and Childhood “G. Barresi”, University of Messina, Messina, Italy

**Keywords:** Migratory locust, Yellow mealworm, Whole house crickets, Frozen formulation, Dried formulation

## Abstract

**Background:**

The European Union (EU) approved the placement on European market of insect-based novel foods. Those foods were defined safe for the consumption for all European population, including children.

**Main body:**

The nutrition committee of the Italian society of Paediatric Hepatology and Nutrition (SIGENP) performed literature research to understand benefits and risk of those use of those NF for Italian children. A special attention was reserved to the European Food Safety Agency (EFSA) reports upon which those novel insect-based were approved.

**Conclusions:**

Based on the current knowledge, despite a possible ecological advantage, the group of expert suggests additional researches before pronouncing on a possible use for children diet, because of insufficient evidence on nutritional benefits and possible food allergies.

## Background

Between February 2022 and February 2023, the European Commission allowed the European Union to place on the market four insect-derived products that are part of the so-called novel foods.

The European Union (EU) defines novel foods (NF) as any food or ingredient that was not used for human consumption on a significant scale in the EU before May 15, 1997 [1].

The four approved insect-based NFs are:

1. Dried yellow mealworm (Tenebrio molitor larva) [2].
2. Frozen and dried Locust (Locusta migratoria) [3] formulations
3. Frozen and dried whole Cricket (Acheta domesticus) formulations [4].
4. Partially defatted Cricket (Acheta domesticus) powder [5].

In order to enable their placing on the market, all these products have been subjected to a safety assessment by the European Food Safety Agency (EFSA).

According to the EU Regulation, the safety assessment should consist of assessing whether the NF is safe under the proposed conditions of use and whether normal consumption of the NF would be nutritionally disadvantageous for the EU population [1]. Thus, no documented negative health effects were found from the EFSA panel form the presence of edible insects in the European food market.

This EFSA assessment does not consider benefit as part of the evaluation. In addition, the analysis of novel foods is not age-specific, but applies to all age groups [6–10].

Given this repositioning on the EU market and the possible use of these products for children, the Nutritional Committee of the Italian Society of Pediatric Gastroenterology and Nutrition (SIGENP) has attempted to summarize available evidence on advantages and disadvantages and to propose an expert opinion on Italian child consumption.

## Main text

### Sustainability

Protein is a fundamental component of human nutrition from birth in order to guarantee tissue building and repairing [11]. Worldwide population growth, increase in income and urbanization determined an increase in animal protein demand [12].

Recent literature has underscored that insect-derived protein can provide a sustainable and ecological substitute for animal protein [13, 14]. Approximately 1900 species of insects have been documented as edible [15]. Many of those species can be directly collected form nature employing very little expenses and thus, their harvesting is suitable also for low-income countries [15]. Another potential benefits derive from the cultivation process of these products, which has been shown to generally use less land and water [16] and result in lower greenhouse gas emissions [16, 17] compared to livestock farming. In addition, with a planet home to 648 million people, 8.4% of the world's population lives in extreme poverty (< $2.15/day) according to the World Bank, the insect market could represent an interesting new market development for farmers in rural communities in developing countries [18, 19] or a new form of business and income for Western countries [20, 21]. For all the above reasons insect food consumption could impact positively the environment.

It is important to highlight that despite current media tendence to use the EFSA term “novel foods” with the meaning of new foods, insect-based foods were used from the ancient Greek [22]. Moreover, insect has always been part of human diet for more than 2 billion inhabitants of Asia, Africa, South America and Oceania [15].

#### Nutritional benefits

**Fig. 1:**
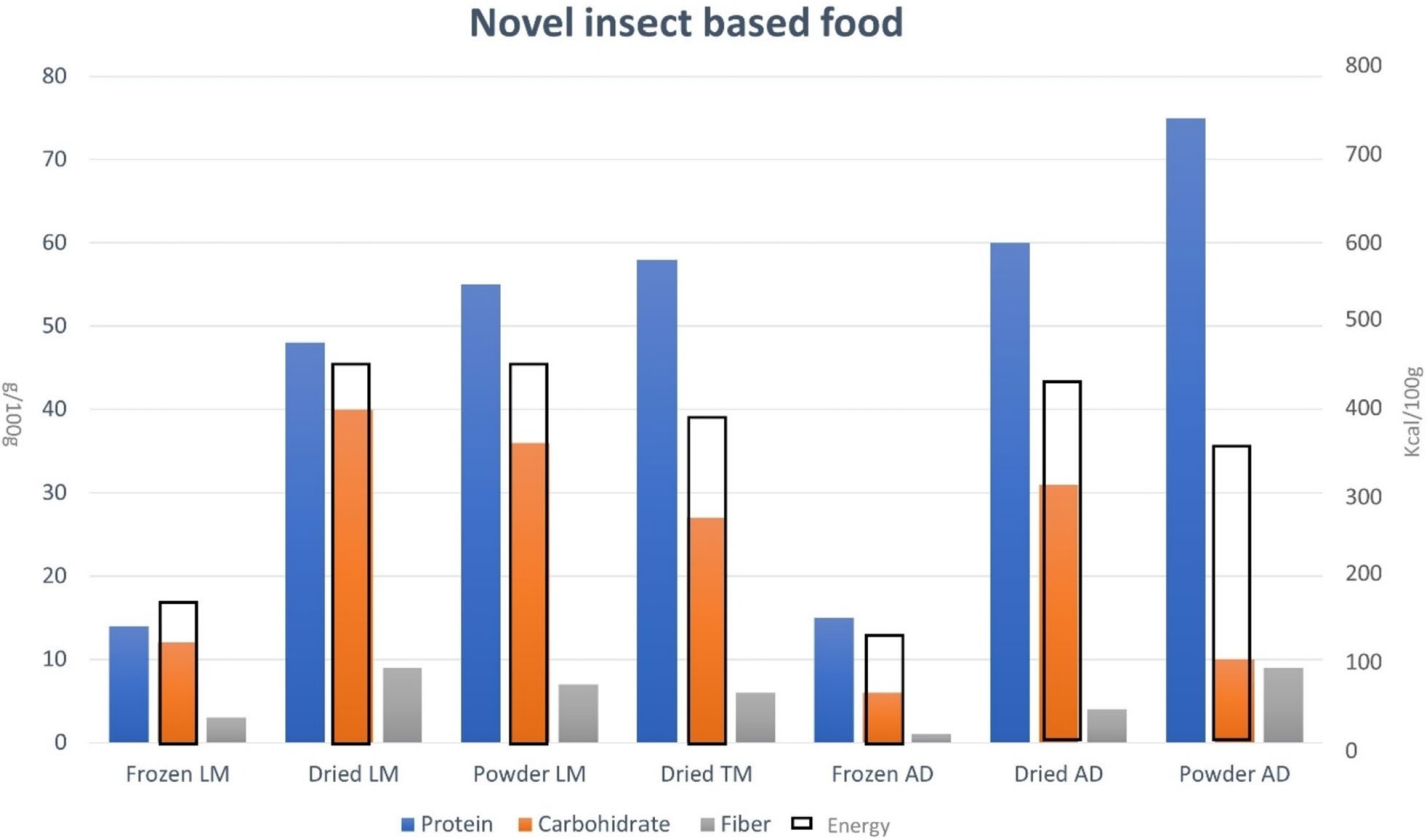
Composition of the novel insect-based foods marketed to date based on EFSA assessments. LM: Locusta Migratoria, TM: Tenebrio Molitor, AD: Acheta Domesticus

Although there is a wide variety of edible insects worldwide, most of them have in common that they consist mainly of high amounts of protein, monounsaturated and polyunsaturated fats and dietary fiber [17]. Analyzing in particular the products that have been approved on the EU market, we have a protein percentage that varies from the lowest 14% for frozen form of migratory locusts or house rickets to the higher level of around 75% for house crickets partially defeated powder. All nutrient contents of the approved products are given in Fig. 1.

Another possible benefit of insect-based food would be supplementation of mineral deficiency like iron deficiency anemia because they are believed to be very rich in iron. If we carefully analyze iron content in the insect feeds approved it varies from 4 to 8 g/100g [6–8, 10]. Furthermore, two recent RCT conducted in young females (18–45 years) reported conflicting results. While mealworm fortification [23] seems to provide a high non heme iron absorption, house rickets [24] showed a low iron absorption rate. No study to date has evaluated this specific topic in children.

Edible insects have always played a major role in oriental medicine because of their potential therapeutic role [25]. A recent publication has further explored the role of bioactive compounds such as polyphenols and flavonoids found in edible insects as antioxidants, anti-inflammatory, antibacterial and insulin regulators [26–28]. The content of polyphenols in insect-based food was recently summarized in a review and varied from 0.3 to 5 g of Gallic Acid Equivalent in 100 g [17]. Up-to date we do not dispose of any publication on the potential anti-inflammatory role of insect-based food in children.

### Nutritional risks

**Fig. 2:**
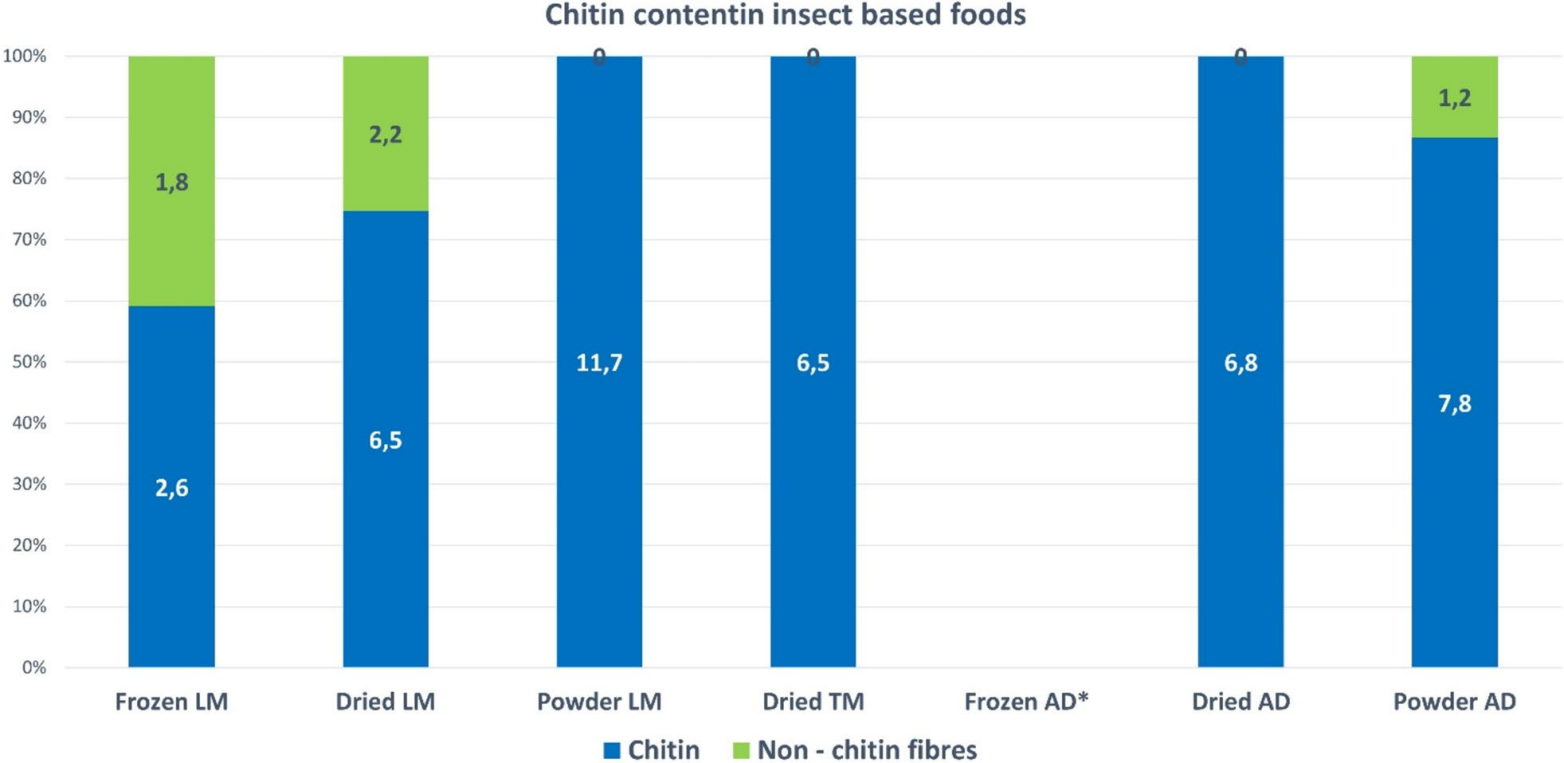
percentage of chitin on total fibers in insect-based NFs based on EFSA assessments. LM: Locusta Migratoria, TM: Tenebrio Molitor, AD: Acheta Domesticus. * data not available

From the analysis of the insect-based food composition, EFSA was able to underline that the main constituent of the fibers of these products is chitin (Fig. 2).

It is a linear polysaccharide composed of b-(1,4)-linked 2-amino-2-deoxy-b-D-glucopyranose and 2-acetamido-2-deoxy-b-D-glucopyranose residues [29]. Chitin poses a problem in estimating the true protein content of NFs. As recently reported by Janssen et al. [30], using the usual nitrogen-to-protein conversion factor of 6.25 overestimates the protein content due to the high non-protein nitrogen, which originates from chitin. A factor in the range of 4.7 to 5.6 depending on the different content in different NFs would be more appropriate with an overestimation of the protein content in the range of 24% and 11% [6–10].

A recent literature review on the subject attempted to summarize the potential effect of edible insects in infant nutritional supplements [31]. A careful selection provided 12 articles originating from Africa (10) and Asia (2), none from western countries. Five of the 12 studies examined the supplemental use of crickets, but only three were published [32–34] and one on migratory locusts [35]; the rest were from non-commercialized insect NF, none from yellow mealworm. The insect-based formulations exceed the recommended daily amount of energy, protein and fat for complementary foods for children aged 6 to 23 months. However, only one [32] of the five studies on rickets analyzed post-intervention nutritional status and found an increase in the prevalence of stunting in the cricket group from 20.7% at baseline to 43.2% after six months of intervention. In addition, Hb and ferritin levels increased between baseline and endline in all groups, including the control group.

### Allergic considerations

Cases of allergic reactions and possible anaphylaxis have been reported as early as 1999 from the use of locusts in China [36] and crickets in Thailand [37]. Recently, the same possible allergic reaction was reported for yellow mealworms [38, 39]. In addition to the direct allergic response described, some studies have highlighted that edible insects may share some of the same allergenic epitopes with other insects such as arthropods, mollusks or nematodes [40, 41]. For all these reasons, EFSA emphasized that ingestion of insect-based NFs authorized on the EU market may trigger a primary allergic reaction or cause cross-reactivity in patients allergic to crustaceans and dusts. In addition, a more thorough analysis of these products by EFSA suggests that possible allergens (particularly gluten) could be part of insect-based feed and thus represent another source of allergens [6–10]. However, it is important to highlight that none of the aforementioned report included pediatric patients, thus, there are no evidence to prohibit those food to atopic/allergic children.

## Conclusion

First of all it is of paramount importance to recognize the very high potential of insect-based novel food as a source of more sustainable proteins in light of the recent shift of paradigm from the lonely healthy diet to the healthy diet from sustainable foods [42]. Furthermore, their potential to prevent starvation and deficiency in low-income countries in unquestionable. However, a word of caution is mandatory when expanding this use to healthy and well-nourished children. In particular because the protein and iron content and absorption of those feeds should be approach with caution and because currently, we lack of any evidence on nutritional benefits of insect-based enriched diet for children at our latitude. Finally, the allergenic potential that can be directly transmitted via cross-reactivity could pose a higher risk for younger children who have not been exposed to other forms of food allergens such as crustaceans or mite dust. However, we should be aware that at our latitude acceptance rate might be low and, when accepted, this will not represent the exclusive protein source of the diet.

In conclusion, despite the security certified by the EFSA panel, some more evidence in children should be produced on nutrients absorption, nutritional benefit and allergic risk, before introducing this “novel food” in children’s diet on a large scale.

## Data Availability

Not applicable.

## Abbreviations

EU: European Union
SIGENP: Italian society of Paediatric Hepatology and Nutrition
EFSA: European Food Safety Agency
NF: Novel foods
